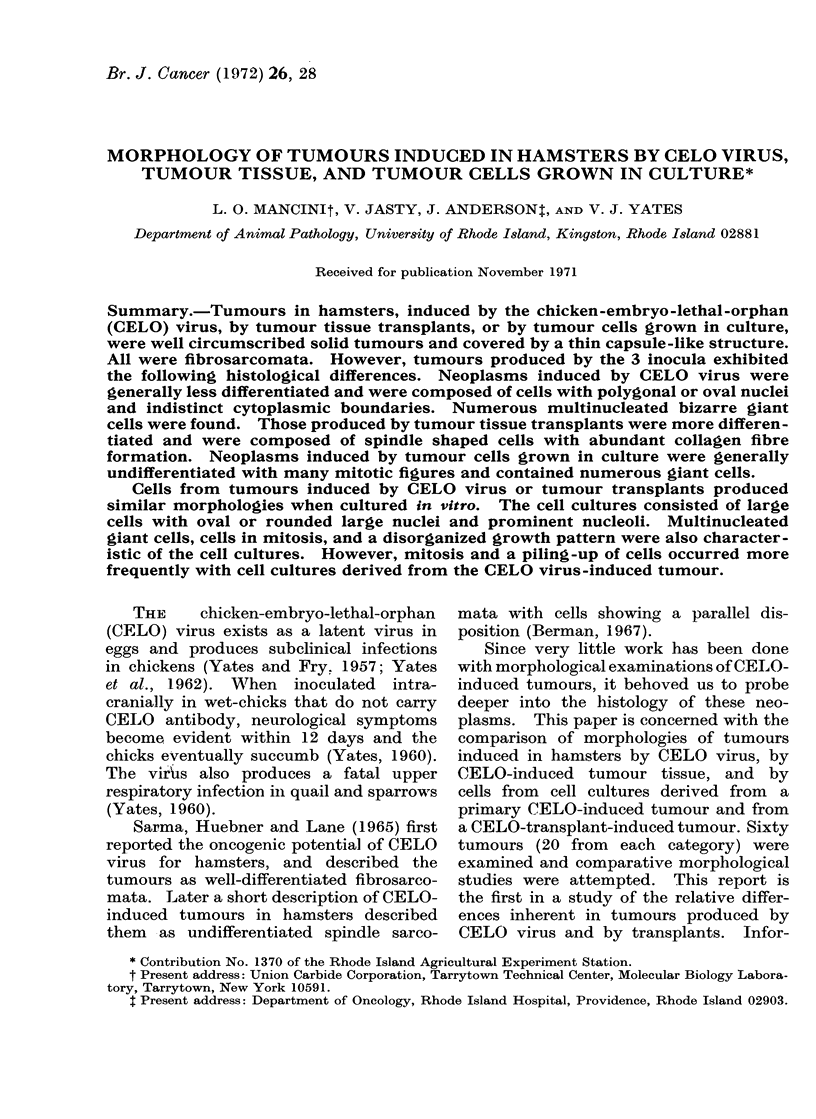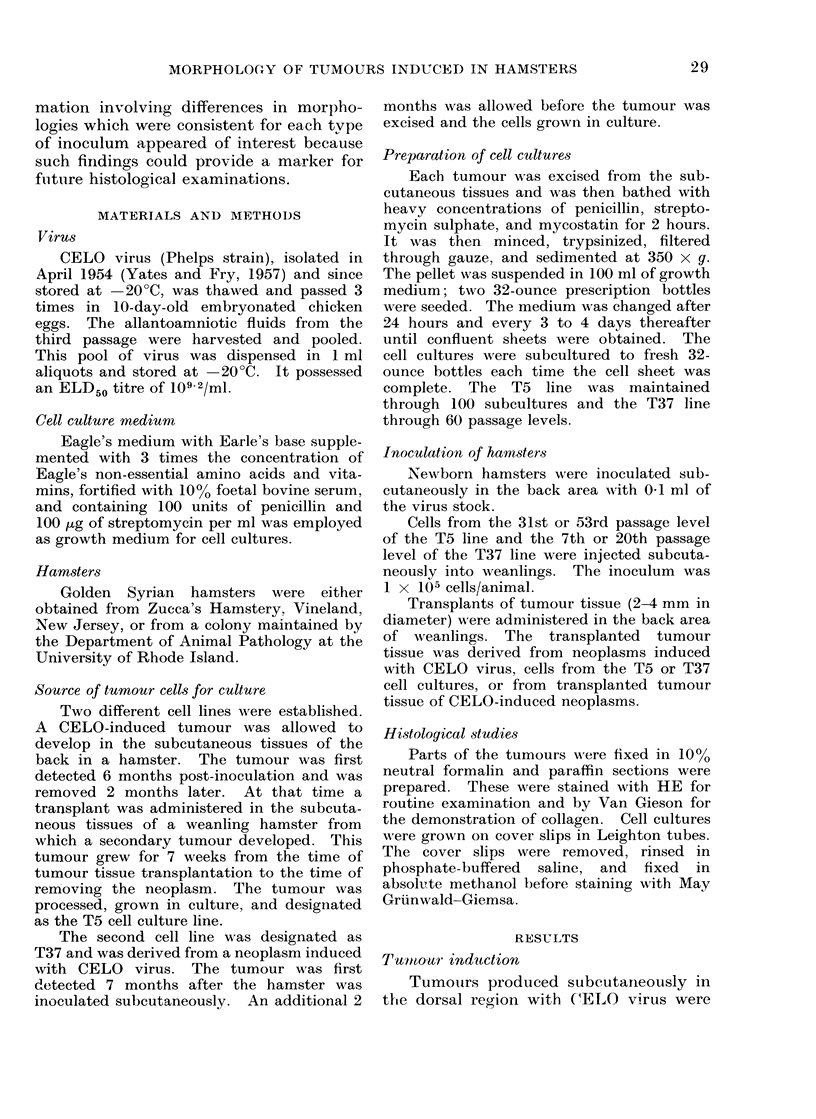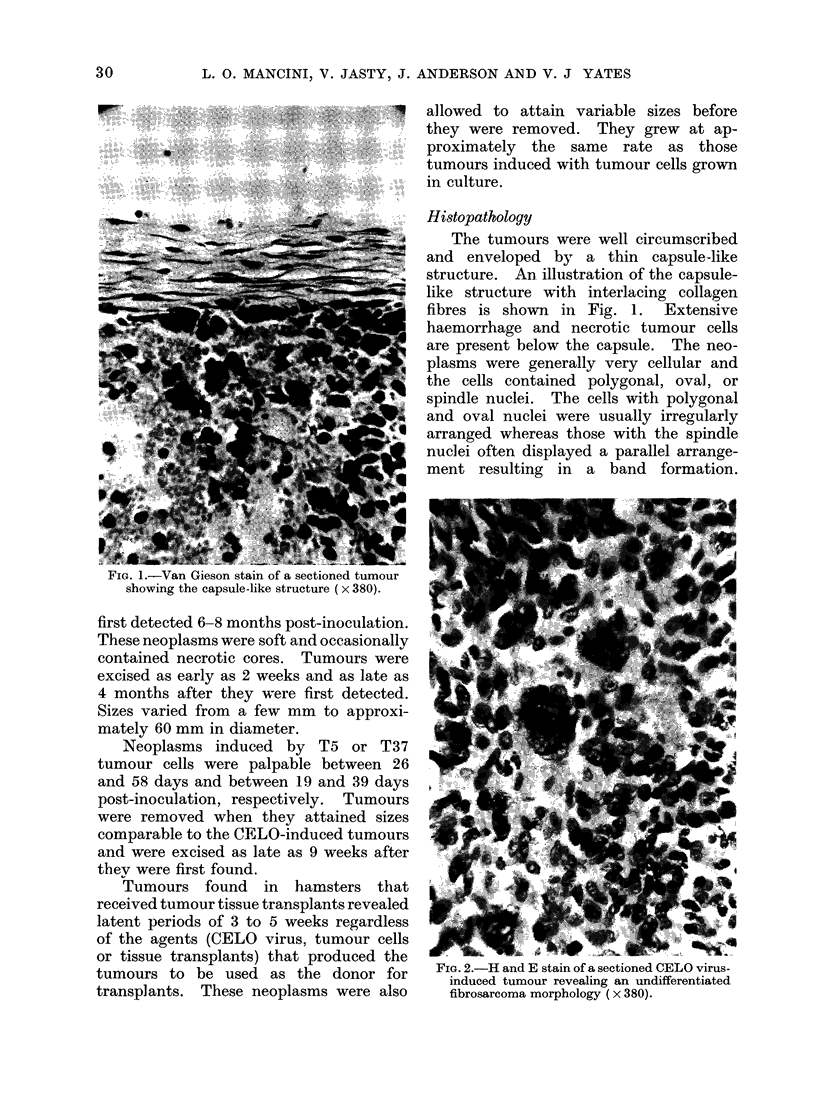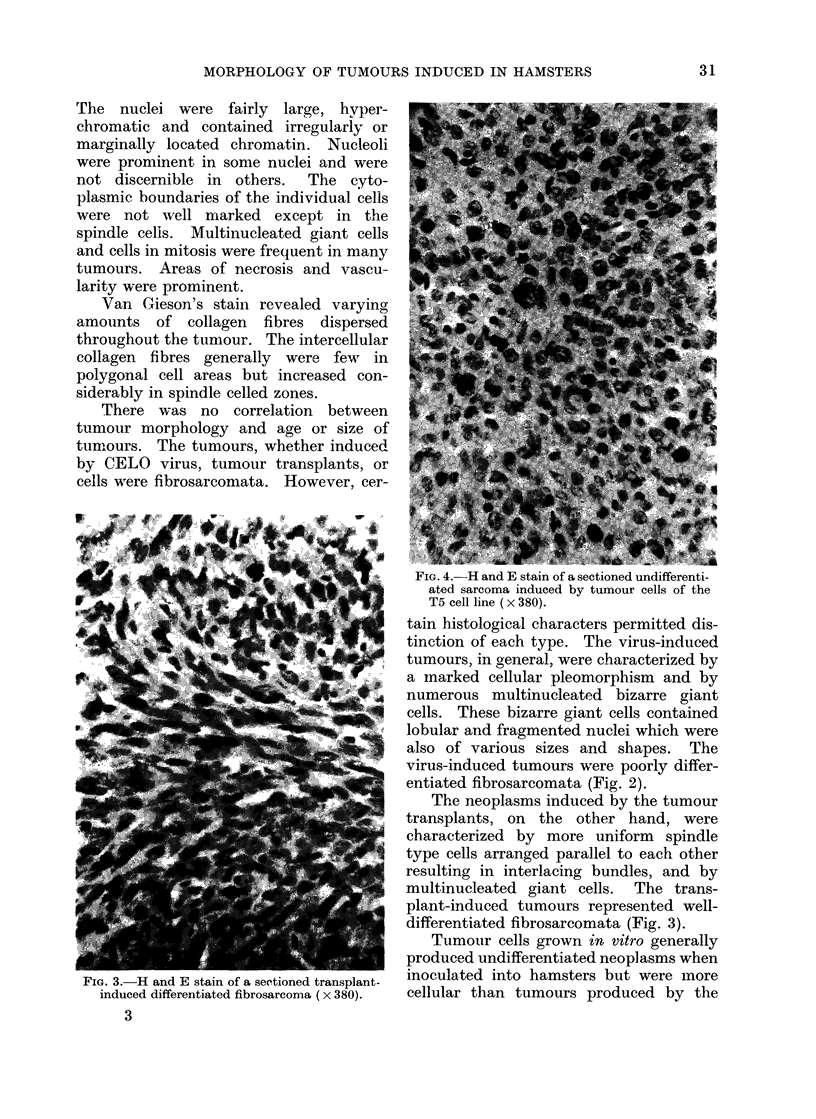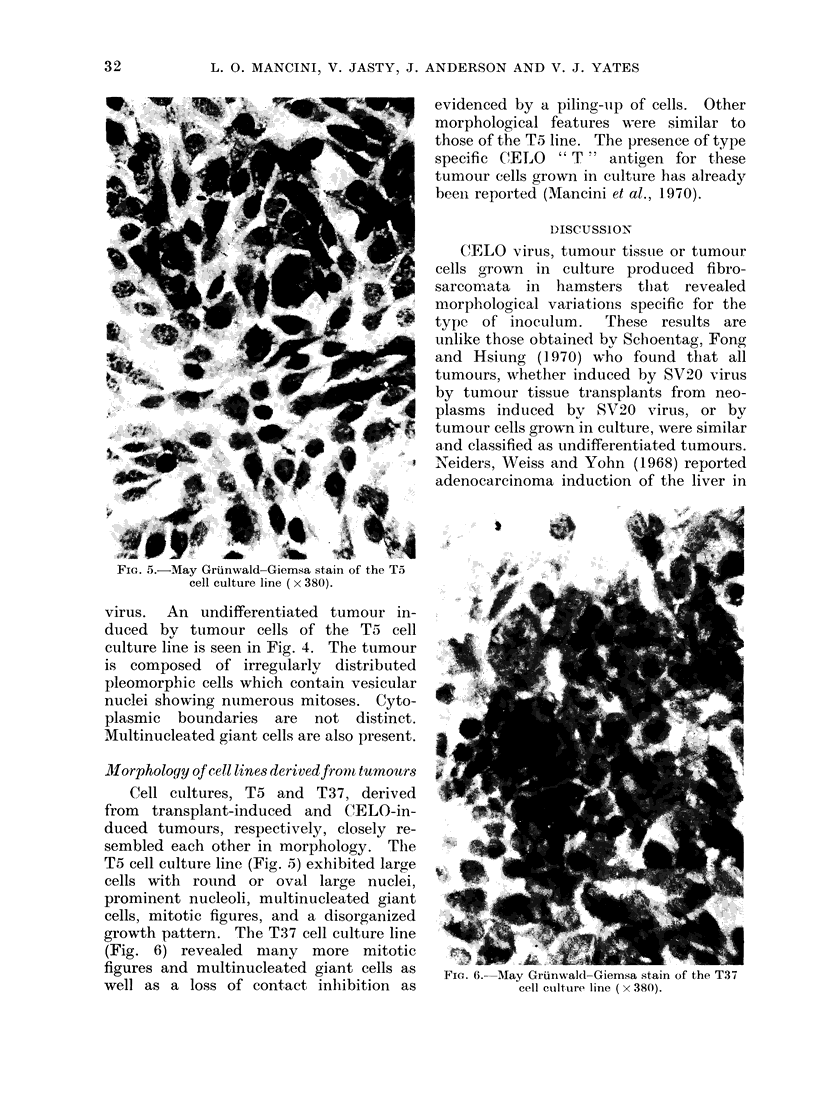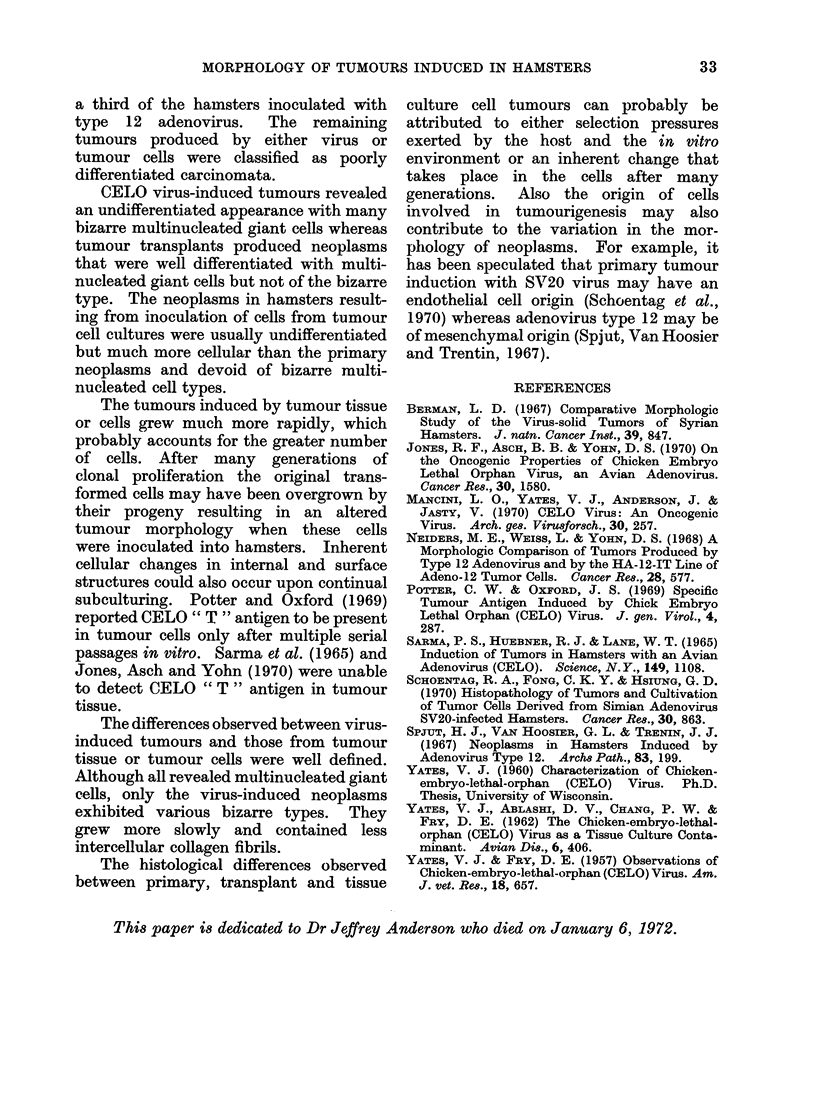# Morphology of Tumours Induced in Hamsters by CELO Virus, Tumour Tissue, and Tumour Cells Grown in Culture*

**DOI:** 10.1038/bjc.1972.6

**Published:** 1972-02

**Authors:** L. O. Mancini, V. Jasty, J. Anderson, V. J. Yates

## Abstract

**Images:**


*Br. J. Cancer* (1972) **26**, 28

### MORPHOLOGY OF TUMOURS INDUCED IN HAMSTERS BY CELO VIRUS, TUMOUR TISSUE, AND TUMOUR CELLS GROWN IN CULTURE*

L. O. MANCINI†, V. JASTY, J. ANDERSON‡, AND V. J. YATES

*Department of Animal Pathology, University of Rhode Island, Kingston, Rhode Island* 02881

Received for publication November 1971

**Summary.—Tumours in hamsters, induced by the chicken-embryo-lethal-orphan (CELO) virus, by tumour tissue transplants, or by tumour cells grown in culture, were well circumscribed solid tumours and covered by a thin capsule-like structure. All were fibrosarcomata. However, tumours produced by the 3 inocula exhibited the following histological differences. Neoplasms induced by CELO virus were generally less differentiated and were composed of cells with polygonal or oval nuclei and indistinct cytoplasmic boundaries. Numerous multinucleated bizarre giant cells were found. Those produced by tumour tissue transplants were more differentiated and were composed of spindle shaped cells with abundant collagen fibre formation. Neoplasms induced by tumour cells grown in culture were generally undifferentiated with many mitotic figures and contained numerous giant cells.**

**Cells from tumours induced by CELO virus or tumour transplants produced similar morphologies when cultured *in vitro*. The cell cultures consisted of large cells with oval or rounded large nuclei and prominent nucleoli. Multinucleated giant cells, cells in mitosis, and a disorganized growth pattern were also characteristic of the cell cultures. However, mitosis and a piling-up of cells occurred more frequently with cell cultures derived from the CELO virus-induced tumour.**

THE chicken-embryo-lethal-orphan (CELO) virus exists as a latent virus in eggs and produces subclinical infections in chickens (Yates and Fry, 1957; Yates *et al.*, 1962). When inoculated intracranially in wet-chicks that do not carry CELO antibody, neurological symptoms become evident within 12 days and the chicks eventually succumb (Yates, 1960). The virus also produces a fatal upper respiratory infection in quail and sparrows (Yates, 1960).

Sarma, Huebner and Lane (1965) first reported the oncogenic potential of CELO virus for hamsters, and described the tumours as well-differentiated fibrosarcomata. Later a short description of CELO-induced tumours in hamsters described them as undifferentiated spindle sarcomata with cells showing a parallel disposition (Berman, 1967).

Since very little work has been done with morphological examinations of CELO-induced tumours, it behoved us to probe deeper into the histology of these neoplasms. This paper is concerned with the comparison of morphologies of tumours induced in hamsters by CELO virus, by CELO-induced tumour tissue, and by cells from cell cultures derived from a primary CELO-induced tumour and from a CELO-transplant-induced tumour. Sixty tumours (20 from each category) were examined and comparative morphological studies were attempted. This report is the first in a study of the relative differences inherent in tumours produced by CELO virus and by transplants. Infor-

* Contribution No. 1370 of the Rhode Island Agricultural Experiment Station.

† Present address: Union Carbide Corporation, Tarrytown Technical Center, Molecular Biology Laboratory, Tarrytown, New York 10591.

‡ Present address: Department of Oncology, Rhode Island Hospital, Providence, Rhode Island 02903.

mation involving differences in morphologies which were consistent for each type of inoculum appeared of interest because such findings could provide a marker for future histological examinations.

### MATERIALS AND METHODS

*Virus*

CELO virus (Phelps strain), isolated in April 1954 (Yates and Fry, 1957) and since stored at −20°C, was thawed and passed 3 times in 10-day-old embryonated chicken eggs. The allantoamniotic fluids from the third passage were harvested and pooled. This pool of virus was dispensed in 1 ml aliquots and stored at −20°C. It possessed an $ELD_{50}$ titre of $10^{9 \cdot 2}$/ml.

*Cell culture medium*

Eagle's medium with Earle's base supplemented with 3 times the concentration of Eagle's non-essential amino acids and vitamins, fortified with 10% foetal bovine serum, and containing 100 units of penicillin and 100 $\mu$g of streptomycin per ml was employed as growth medium for cell cultures.

*Hamsters*

Golden Syrian hamsters were either obtained from Zucca's Hamstery, Vineland, New Jersey, or from a colony maintained by the Department of Animal Pathology at the University of Rhode Island.

*Source of tumour cells for culture*

Two different cell lines were established. A CELO-induced tumour was allowed to develop in the subcutaneous tissues of the back in a hamster. The tumour was first detected 6 months post-inoculation and was removed 2 months later. At that time a transplant was administered in the subcutaneous tissues of a weanling hamster from which a secondary tumour developed. This tumour grew for 7 weeks from the time of tumour tissue transplantation to the time of removing the neoplasm. The tumour was processed, grown in culture, and designated as the T5 cell culture line.

The second cell line was designated as T37 and was derived from a neoplasm induced with CELO virus. The tumour was first detected 7 months after the hamster was inoculated subcutaneously. An additional 2 months was allowed before the tumour was excised and the cells grown in culture.

*Preparation of cell cultures*

Each tumour was excised from the subcutaneous tissues and was then bathed with heavy concentrations of penicillin, streptomycin sulphate, and mycostatin for 2 hours. It was then minced, trypsinized, filtered through gauze, and sedimented at 350 × *g*. The pellet was suspended in 100 ml of growth medium; two 32-ounce prescription bottles were seeded. The medium was changed after 24 hours and every 3 to 4 days thereafter until confluent sheets were obtained. The cell cultures were subcultured to fresh 32-ounce bottles each time the cell sheet was complete. The T5 line was maintained through 100 subcultures and the T37 line through 60 passage levels.

*Inoculation of hamsters*

Newborn hamsters were inoculated subcutaneously in the back area with 0·1 ml of the virus stock.

Cells from the 31st or 53rd passage level of the T5 line and the 7th or 20th passage level of the T37 line were injected subcutaneously into weanlings. The inoculum was $1 \times 10^5$ cells/animal.

Transplants of tumour tissue (2–4 mm in diameter) were administered in the back area of weanlings. The transplanted tumour tissue was derived from neoplasms induced with CELO virus, cells from the T5 or T37 cell cultures, or from transplanted tumour tissue of CELO-induced neoplasms.

*Histological studies*

Parts of the tumours were fixed in 10% neutral formalin and paraffin sections were prepared. These were stained with HE for routine examination and by Van Gieson for the demonstration of collagen. Cell cultures were grown on cover slips in Leighton tubes. The cover slips were removed, rinsed in phosphate-buffered saline, and fixed in absolute methanol before staining with May Grünwald–Giemsa.

### RESULTS

*Tumour induction*

Tumours produced subcutaneously in the dorsal region with CELO virus were

FIG. 1.—Van Gieson stain of a sectioned tumour showing the capsule-like structure ($\times$380).

first detected 6–8 months post-inoculation. These neoplasms were soft and occasionally contained necrotic cores. Tumours were excised as early as 2 weeks and as late as 4 months after they were first detected. Sizes varied from a few mm to approximately 60 mm in diameter.

Neoplasms induced by T5 or T37 tumour cells were palpable between 26 and 58 days and between 19 and 39 days post-inoculation, respectively. Tumours were removed when they attained sizes comparable to the CELO-induced tumours and were excised as late as 9 weeks after they were first found.

Tumours found in hamsters that received tumour tissue transplants revealed latent periods of 3 to 5 weeks regardless of the agents (CELO virus, tumour cells or tissue transplants) that produced the tumours to be used as the donor for transplants. These neoplasms were also allowed to attain variable sizes before they were removed. They grew at approximately the same rate as those tumours induced with tumour cells grown in culture.

*Histopathology*

The tumours were well circumscribed and enveloped by a thin capsule-like structure. An illustration of the capsule-like structure with interlacing collagen fibres is shown in Fig. 1. Extensive haemorrhage and necrotic tumour cells are present below the capsule. The neoplasms were generally very cellular and the cells contained polygonal, oval, or spindle nuclei. The cells with polygonal and oval nuclei were usually irregularly arranged whereas those with the spindle nuclei often displayed a parallel arrangement resulting in a band formation.

FIG. 2.—H and E stain of a sectioned CELO virus-induced tumour revealing an undifferentiated fibrosarcoma morphology ($\times$380).

The nuclei were fairly large, hyperchromatic and contained irregularly or marginally located chromatin. Nucleoli were prominent in some nuclei and were not discernible in others. The cytoplasmic boundaries of the individual cells were not well marked except in the spindle cells. Multinucleated giant cells and cells in mitosis were frequent in many tumours. Areas of necrosis and vascularity were prominent.

Van Gieson's stain revealed varying amounts of collagen fibres dispersed throughout the tumour. The intercellular collagen fibres generally were few in polygonal cell areas but increased considerably in spindle celled zones.

There was no correlation between tumour morphology and age or size of tumours. The tumours, whether induced by CELO virus, tumour transplants, or cells were fibrosarcomata. However, certain histological characters permitted distinction of each type. The virus-induced tumours, in general, were characterized by a marked cellular pleomorphism and by numerous multinucleated bizarre giant cells. These bizarre giant cells contained lobular and fragmented nuclei which were also of various sizes and shapes. The virus-induced tumours were poorly differentiated fibrosarcomata (Fig. 2).

Fig. 3.—H and E stain of a sectioned transplant-induced differentiated fibrosarcoma ($\times$ 380).

Fig. 4.—H and E stain of a sectioned undifferentiated sarcoma induced by tumour cells of the T5 cell line ($\times$ 380).

The neoplasms induced by the tumour transplants, on the other hand, were characterized by more uniform spindle type cells arranged parallel to each other resulting in interlacing bundles, and by multinucleated giant cells. The transplant-induced tumours represented well-differentiated fibrosarcomata (Fig. 3).

Tumour cells grown *in vitro* generally produced undifferentiated neoplasms when inoculated into hamsters but were more cellular than tumours produced by the

FIG. 5.—May Grünwald–Giemsa stain of the T5 cell culture line (×380).

virus. An undifferentiated tumour induced by tumour cells of the T5 cell culture line is seen in Fig. 4. The tumour is composed of irregularly distributed pleomorphic cells which contain vesicular nuclei showing numerous mitoses. Cytoplasmic boundaries are not distinct. Multinucleated giant cells are also present.

*Morphology of cell lines derived from tumours*

Cell cultures, T5 and T37, derived from transplant-induced and CELO-induced tumours, respectively, closely resembled each other in morphology. The T5 cell culture line (Fig. 5) exhibited large cells with round or oval large nuclei, prominent nucleoli, multinucleated giant cells, mitotic figures, and a disorganized growth pattern. The T37 cell culture line (Fig. 6) revealed many more mitotic figures and multinucleated giant cells as well as a loss of contact inhibition as evidenced by a piling-up of cells. Other morphological features were similar to those of the T5 line. The presence of type specific CELO " T " antigen for these tumour cells grown in culture has already been reported (Mancini *et al.*, 1970).

### DISCUSSION

CELO virus, tumour tissue or tumour cells grown in culture produced fibrosarcomata in hamsters that revealed morphological variations specific for the type of inoculum. These results are unlike those obtained by Schoentag, Fong and Hsiung (1970) who found that all tumours, whether induced by SV20 virus by tumour tissue transplants from neoplasms induced by SV20 virus, or by tumour cells grown in culture, were similar and classified as undifferentiated tumours. Neiders, Weiss and Yohn (1968) reported adenocarcinoma induction of the liver in

FIG. 6.—May Grünwald–Giemsa stain of the T37 cell culture line (×380).

a third of the hamsters inoculated with type 12 adenovirus. The remaining tumours produced by either virus or tumour cells were classified as poorly differentiated carcinomata.

CELO virus-induced tumours revealed an undifferentiated appearance with many bizarre multinucleated giant cells whereas tumour transplants produced neoplasms that were well differentiated with multinucleated giant cells but not of the bizarre type. The neoplasms in hamsters resulting from inoculation of cells from tumour cell cultures were usually undifferentiated but much more cellular than the primary neoplasms and devoid of bizarre multinucleated cell types.

The tumours induced by tumour tissue or cells grew much more rapidly, which probably accounts for the greater number of cells. After many generations of clonal proliferation the original transformed cells may have been overgrown by their progeny resulting in an altered tumour morphology when these cells were inoculated into hamsters. Inherent cellular changes in internal and surface structures could also occur upon continual subculturing. Potter and Oxford (1969) reported CELO " T " antigen to be present in tumour cells only after multiple serial passages *in vitro*. Sarma *et al.* (1965) and Jones, Asch and Yohn (1970) were unable to detect CELO " T " antigen in tumour tissue.

The differences observed between virus-induced tumours and those from tumour tissue or tumour cells were well defined. Although all revealed multinucleated giant cells, only the virus-induced neoplasms exhibited various bizarre types. They grew more slowly and contained less intercellular collagen fibrils.

The histological differences observed between primary, transplant and tissue culture cell tumours can probably be attributed to either selection pressures exerted by the host and the *in vitro* environment or an inherent change that takes place in the cells after many generations. Also the origin of cells involved in tumourigenesis may also contribute to the variation in the morphology of neoplasms. For example, it has been speculated that primary tumour induction with SV20 virus may have an endothelial cell origin (Schoentag *et al.*, 1970) whereas adenovirus type 12 may be of mesenchymal origin (Spjut, Van Hoosier and Trentin, 1967).

### REFERENCES

Berman, L. D. (1967) Comparative Morphologic Study of the Virus-solid Tumors of Syrian Hamsters. *J. natn. Cancer Inst.*, **39**, 847.

Jones, R. F., Asch, B. B. & Yohn, D. S. (1970) On the Oncogenic Properties of Chicken Embryo Lethal Orphan Virus, an Avian Adenovirus. *Cancer Res.*, **30**, 1580.

Mancini, L. O., Yates, V. J., Anderson, J. & Jasty, V. (1970) CELO Virus: An Oncogenic Virus. *Arch. ges. Virusforsch.*, **30**, 257.

Neiders, M. E., Weiss, L. & Yohn, D. S. (1968) A Morphologic Comparison of Tumors Produced by Type 12 Adenovirus and by the HA-12-IT Line of Adeno-12 Tumor Cells. *Cancer Res.*, **28**, 577.

Potter, C. W. & Oxford, J. S. (1969) Specific Tumour Antigen Induced by Chick Embryo Lethal Orphan (CELO) Virus. *J. gen. Virol.*, **4**, 287.

Sarma, P. S., Huebner, R. J. & Lane, W. T. (1965) Induction of Tumors in Hamsters with an Avian Adenovirus (CELO). *Science, N.Y.*, **149**, 1108.

Schoentag, R. A., Fong, C. K. Y. & Hsiung, G. D. (1970) Histopathology of Tumors and Cultivation of Tumor Cells Derived from Simian Adenovirus SV20-infected Hamsters. *Cancer Res.*, **30**, 863.

Spjut, H. J., Van Hoosier, G. L. & Trenin, J. J. (1967) Neoplasms in Hamsters Induced by Adenovirus Type 12. *Archs Path.*, **83**, 199.

Yates, V. J. (1960) Characterization of Chicken-embryo-lethal-orphan (CELO) Virus. Ph.D. Thesis, University of Wisconsin.

Yates, V. J., Ablashi, D. V., Chang, P. W. & Fry, D. E. (1962) The Chicken-embryo-lethal-orphan (CELO) Virus as a Tissue Culture Contaminant. *Avian Dis.*, **6**, 406.

Yates, V. J. & Fry, D. E. (1957) Observations of Chicken-embryo-lethal-orphan (CELO) Virus. *Am. J. vet. Res.*, **18**, 657.

*This paper is dedicated to Dr Jeffrey Anderson who died on January 6, 1972.*